# Aging is associated with increased collagen type IV accumulation in the basal lamina of human cerebral microvessels

**DOI:** 10.1186/1471-2202-5-37

**Published:** 2004-09-24

**Authors:** Olga Uspenskaia, Martin Liebetrau, Jochen Herms, Adrian Danek, Gerhard F Hamann

**Affiliations:** 1 Department of Neurology, Klinikum Grosshadern, Ludwig-Maximilians-University, Munich, Germany; 2 Department of Neuropathology, Klinikum Grosshadern, Ludwig-Maximilians-University, Munich, Germany

## Abstract

**Background:**

Microvascular alterations contribute to the development of stroke and vascular dementia. The goal of this study was to evaluate age and hypertension related changes of the basal lamina in cerebral microvessels of individuals, who died from non-cerebral causes.

**Results:**

We examined 27 human brains: 11 young and 16 old patients. Old patients were divided into two subgroups, those with hypertension (n = 8) and those without hypertension (n = 8). Basal lamina changes of the cerebral microvessels were determined in the putamen using antibodies against collagen type IV and by quantitative analysis of vessel number, total stained area of collagen, thickness of the vessel wall and lumen, and relative staining intensity using immunofluorescence. The total number of collagen positive vessels per microscopic field was reduced in old compared to young subjects (12.0+/-0.6 vs. 15.1+/-1.2, p = 0.02). The relative collagen content per vessel (1.01+/-0.06 vs. 0.76+/-0.05, p = 0.01) and the relative collagen intensity (233.1+/-4.5 vs. 167.8+/-10.6, p < 0.0001) shown by immunofluorescence were higher in the older compared to the younger patients with a consecutive reduction of the lumen / wall ratio (1.29+/-0.05 vs. 3.29+/-0.15, p < 0.0001). No differences were observed for these parameters between old hypertensive and non-hypertensive patients.

**Conclusions:**

The present data show age-related changes of the cerebral microvessels in sections of human putamen for the first time. Due to the accumulation of collagen, microvessels thicken and show a reduction in their lumen. Besides this, the number of vessels decreases. These findings might represent a precondition for the development of vascular cognitive impairment. However, hypertension was not proven to modulate these changes.

## Background

Aging is associated with a deterioration of cognitive function including a decrease in the ability to process and store new information [1]. Processes that might negatively affect cognitive function during aging are manifold. Among them, the cerebral vascular system has a major impact on brain function. Craigie first described that the density of cerebral microvessels may correlate with functional activity [2]. However, morphological studies of the microvessels have been inconsistent up to now. Meier-Ruge and coworkers reported an increase in capillary density in older individuals [3], whereas others had shown a reduction [4]. One important reason for the different findings in microvascular changes might be the heterogeneity of the examined brain regions between, but also within the studies. Nevertheless changes in the cerebral microvessels have a major impact on secondary pathophysiological changes, like reduced cerebral blood flow (CBF) [5,6], and a decrease in doppler sonographic blood flow velocity in old age [7]. In addition, microvascular alterations are responsible for reduced cerebral metabolic rates for oxygen and cerebral glucose utilization, which is observed with increasing age [8,9] leading to an impaired transport of nutrients which in turn impairs neuronal function. Additional factors such as chronic hypertension may accelerate the progression of age-related capillary changes [10].

Although changes of cerebral vessels with aging and hypertension have been reported, there are presently no consistent data on the microvascular basal lamina. The aim of our study was an evaluation of age-related changes on cerebral microvessels and on the vascular extracellular matrix in humans and the possible impact of chronic hypertension using several different immunohistochemical methods for the detection of collagen type IV.

## Results

### Determination of collagen type IV in microvessels by immunohistochemistry

Old patients (OP) showed 12.0 +/- 0.6 vessels per microscopic field, and young patients (YP) exhibited 15.1 ± 1.2 vessels per microscopic field (p = 0.02). The total area of collagen type IV positive vessels did not differ between OP and YP (11.0 ± 0.5 vs. 11.4 ± 0.6, n.s.). Therefore the calculated content of collagen type IV per vessel was higher in OP than in YP (1.01 ± 0.06 vs. 0.76 ± 0.05, p = 0.01). No differences were observed between old non hypertensive patients (ONHP) and old hypertensive patients (OHP) (vessels per microscopic field: 11.3 ± 1.1 in ONHP vs. 12.7 ± 0.7 in OHP, n.s.; area of collagen type IV: 12.1 ± 0.8 in ONHP vs. 10.6 ± 0.9 in OHP, n.s.; calculated content of collagen type IV per vessel: 1.11 ± 0.1 in ONHP vs. 0.91 ± 0.07 in OHP, n.s.).

### Thickness of vessel wall and lumen

**Figure 1:**
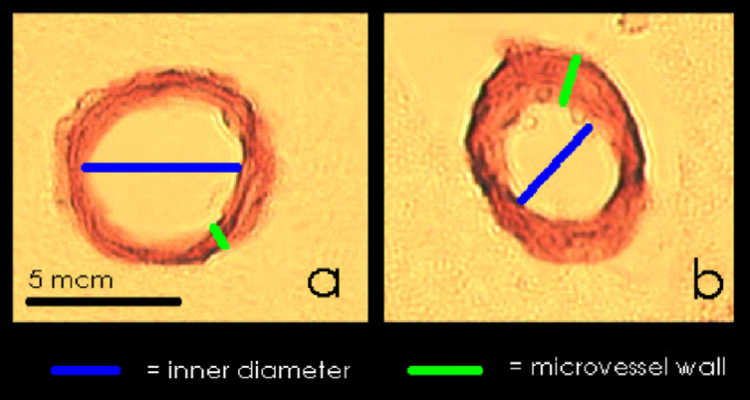
**Analysis of the thickness of the vessel wall and the inner diameter of the vessel lumen in YP and OP. **Figure A indicates YP, Figure B indicates OP.

**Figure 2:**
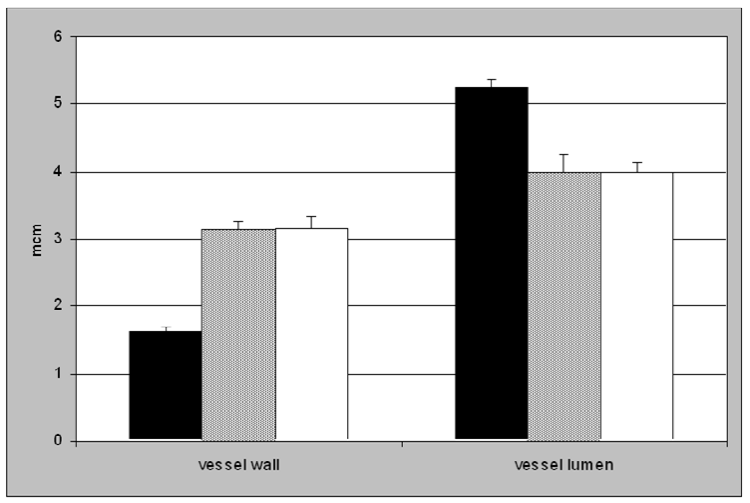
**Analysis of the thickness of the vessel wall and the inner diameter of the vessel lumen. **Black boxes indicate YP. OP were divided into ONHP (grey boxes), and OHP (white boxes). The YP group revealed a significant thinner vessel wall and a larger vessel lumen than OP (p < 0.0001). No difference was observed between ONHP and OHP.

Microvessel wall thickness, inner lumen and ratio lumen/wall thickness were statistically different in OP vs. YP. The vessel wall was thicker in old than in young patients (3.14 ± 0.10 μm vs. 1.62 ± 0.06 μm, p < 0.0001). In addition the vessel lumen was reduced in the old group compared to the young group (4.00 ± 0.14 μm vs. 5.24 ± 0.13 μm, p < 0.0001, Figure 1 and 2). Therefore the ratio between the thickness of the vessel lumen and the vessel wall was lowered in the OP group compared to YP (1.29 ± 0.05 vs. 3.29 ± 0.15, p < 0.0001). The comparison of the same parameters in ONHP vs. OHP showed no significant distinctions (thickness of vessel wall: 3.13 ± 0.12 μm in ONHP vs. 3.16 ± 0.18 μm in OHP, n.s.; vessel lumen: 4.00 ± 0.26 μm in ONHP vs. 4.00 ± 0.14 μm in OHP, n.s.; ratio: 1.28 ± 0.07 in ONHP vs. 1.30 ± 0.08 in OHP, n.s.)

### Relative collagen type IV intensity in microvessels determined by CLSM

**Figure 3:**
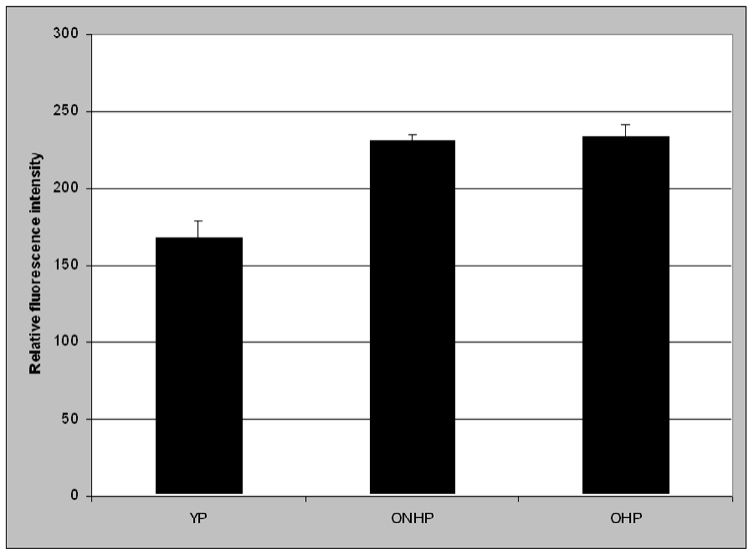
**Analysis of the relative collagen type IV content in the microvessels by CLSM. **The difference was significant between YP and OP (not shown), but not between ONHP and OHP.

Analysis of the relative intensity of collagen type-IV positive vessels was performed by confocal laser scanning microscopy. The relative content of collagen type IV in the microvessel wall was higher in OP than in YP (233.1 ± 4.5 vs. 167.8 ± 10.6, p < 0.0001). Again, the intensity in non-hypertensive old persons showed no difference compared to hypertensive old patients (230.7 ± 4.2 vs. 234.3 ± 6.7, n.s., Figure 3).

## Discussion

In the present study, we investigated age- and hypertension-related alterations in the vascular extracellular matrix and the basal lamina in human brains. Our main finding is the age-related change of the basal lamina component collagen type IV. In old as compared to young individuals we found a significant decrease of the vessel number containing collagen type IV, a thickening of the vessel wall and narrowing of the vessel lumen and an increase in collagen type IV content per vessel. These changes in extracellular matrix proteins were demonstrated by immunohistochemistry as well as by confocal laser scanning microscopy. However, we were unable to establish significant changes of the microvessels in hypertensive old persons compared to normotensive old patients.

These results are in good accordance with previous studies that have shown basal lamina thickening in experimental studies [10-12]. Research on basal lamina changes in humans has mostly been focused on their relationship to neurodegeneration. In Alzheimer's patients Kalaria and coworkers found a 55% increase of collagen type IV content in cerebral microvessels in comparison to age-matched controls [13]. Farkas and colleagues described collagen accumulation in the basal lamina of Parkinson's disease [14]. Data about changes in the cerebral microvasculature in normal aging, however, are scarce. One study examined cerebral microvessels in human neocortex, yet failed to demonstrate an age-related thickening of the basal lamina [15], however one study was able to show a decrease of microvascular density by age in the hypothalamus [4], interestingly also this study failed to show hypertension related changes. One explanation for the decreased microvascular density in the putamen might be a reduced neoangiogenesis in the aging human brain, this hypothesis should be examined in further studies.

One reason for the inconclusive findings in human cerebral microvessels might be the local origin of the samples, as up to now rarefaction was only found in the deep grey matter. The microvasculature is organized differently in the basal ganglia than in the cortex. The basal ganglia microvasculature has the geometry of a tree-like vascular bed, in contrast the cortical microvascular networks are rather organized like a grid structure [16]. Due to this reason the deep grey matter might be more vulnerable to metabolic changes in age, with reduced capabilities for neoangiogenesis, as this might compensatory occur in cortical areas. Therefore differential patterns of microvascular changes within the brain might occur, resulting in controversial results of microvascular density in the aging brain. Another explanation seems to be a methodological one. As the region of interest in putaminal sections can be exactly defined from section to section, analyses of cortical areas might be more problematic resulting in an imprecise definition of comparable areas [15].

The reasons for the alterations of vascular extracellular matrix proteins in aging are largely unknown. Not only age is associated with an increase of extracellular matrix proteins, as these changes were observed in several diseases, like hypertension, brain tumors [17], HIV-encephalopathy [18], Alzheimer's [13] or Parkinson's disease [14]. One explanation for the extracellular matrix accumulation might be the reduction of the proteolytic systems activity. The matrix metalloproteinases (MMP) and the natural tissue endogenous inhibitors (TIMP), as well as the plasminogen/plasmin system are involved in the regulation of ECM metabolism [19,20], and changes in these proteases activity may contribute to vascular remodelling in age by modulating the extracellular matrix components. Therefore a reduction of these proteases might result in a decreased turnover of the basal lamina with a consecutive increase of these components. The reduction of MMP activity by age was recently shown in an experimental study [21] and in humans by antihypertensive treatment [22], however studies about the role of these proteases in the aging human brain are lacking and the impact has to be evaluated in further studies.

The strength of our study is the combination of several different methods for the detection of basal lamina changes. Even if the study by Abernethy and colleagues [4] was the first one, that showed age related changes in the deep grey matter, their study has some limitations. First they used a nonspecific staining technique with alkaline phosphatase, and second no detailed morphometric analysis of the vessels or changes of the basal lamina were performed. On the other hand, previously published studies most often used only one method for the determination of microvascular changes. For example the analyses of the vessel wall and lumen was widely used as the only parameter. [23-25] Unquestionably this method has a subjective approach and therefore we see our results of the increased vessel wall/lumen ratio in aging as a confirmation of previous results. But in contrast to these studies our approach employed an additional variety of complementary methods to examine the increase of basal membrane components: number of vessels, total stained area of collagen type IV, relative collagen type IV content per vessel and relative immunofluorescence by CLSM, all indicating into the same direction.

Some methodological issues have to be discussed, starting with the unexpected lack of a difference between old normotensive and old hypertensive patients. One explanation might be due to silent hypertension in the ONHP group, as well as treated hypertension in the OHP group. We tried to minimize this problem due to carefully study of the case records in all patients. In addition in our study hypertension was defined as a history of hypertension, rather than the actual blood pressure in hospital, as these patients with severe diseases might not had representative blood pressure values in their last days or weeks of life than decades before. On the other hand another study failed to show the impact of hypertension on microvascular densitiy in a neuropathological study of the human hypothalamus [4]. This unexpected lack of hypertension related changes in the microvessels should be regarded as preliminary, as neither the duration of hypertension nor the effectiveness of treatment was considered.

## Conclusions

The present data show age-related changes of the cerebral microvessels in sections of human putamen for the first time. Due to the accumulation of collagen, microvessels thicken and show a reduction in their lumen. Besides this, the number of vessels decreases. These findings might represent a precondition for the development of vascular cognitive impairment.

## Methods

The study was performed on 27 post-mortem human brain samples from the putamen, which were taken from autopsy. The clinical diagnoses were confirmed by routine pathology and are shown in the table. Two groups of subjects were compared: first young patients, all without a history of hypertension (YP; n = 11, mean age 38.8 ± 6.8 years), and old patients (OP, n = 16, mean age 73.9 ± 4.1 years). The old patients were divided into two subgroups, those without a history of hypertension (ONHP, n = 8, mean age 73.1 ± 4.9 years), and those with a history of hypertension (OHP, n = 8, mean age 74.6 ± 3.4 years). There were no significant differences in age between OHP and ONHP and in sex between YP and OP, as well as between ONHP and OHP (see Table 1).

The putamen either of the right or left side were removed completely and fixated in paraffin. We chose the putamen region, as it is easily to define and vascular changes and strokes are predominantly located in this area. The blocks were cut cross sectional in the same anterior-posterior direction resulting in axial sections with a thickness of 10 μm. The sections were deparaffinized and immersed at 37°C in 0.4% Pepsin (Sigma, Germany) in 0.01 N HCl for one hour. Collagen IV-positive vessels were stained with a monoclonal mouse anti-collagen-IV antibody (Sigma, Germany). Each section was incubated with 150 μl of the primary antibody solution (at a concentration of 1:200) for two hours at 37°C followed by incubation with biotinylated secondary antibody against mouse IgG for 30 minutes at 37°C (Vector Laboratories). Vectastain ABC reagent was added for 30 minutes at 37°C. Chromogen (AEC Kit Biomeda Corp.) was used to develop the peroxidase signal. Negative and positive controls were routinely performed in each staining experiment. The same procedure was used for immunofluorescence staining. Instead of using the Vectastain ABC kit containing avidin, avidin marked FITC (Dianova, Hamburg, Germany) was added for 30 minutes at a dilution of 1:100.

The number of peroxidase stained vessels was determined with the aid of a computerized video imaging system at a magnification of ×100 (Optimas Version 6.5 from Media Cybernetics, Silver Spring, USA). Only vessels smaller than 30 μm were included. Total area of collagen IV positive vessels in the sections was analyzed using the same system. Results are presented in arbitrary units. To obtain the relative amount of collagen type IV per vessel the area of collagen type IV was divided by the number of stained vessels per microscopic field ([collagen type IV/microscopic field]/[vessels/microscopic field]) The size of the observed microscopic field was 150 × 200 μm.

To estimate microvessel hypertrophy, the ratio between the diameter of vessel lumen and vessel wall, respectively, was calculated semiquantitatively with the help of a second computerized video imaging system (Medmo, Homburg, Germany). Twenty entire cross-sectional microvessels from the putamen were randomly selected at a magnification of ×400. To calculate the wall to lumen ratio, average distances of vessel wall and vessel lumen were selected.

Fluorescence intensity measurements of microvessel-associated FITC anti-mouse IgG against the anti-collagen antibody were performed with confocal laser scanning microscopy (CLSM, Leica, Heidelberg, Germany). All measurements were performed with the same pinhole size, brightness and contrast, zoom, and laser time. Each vessel was scanned in the *z *plane (10 scans per 1 μm), and a summed image was calculated. Also, a summed image was obtained from the background area to normalize the local intensity to the background. The normalized intensity is expressed as mean ± SEM for each microvessel using a scale from 0 to 255 arbitrary units (U). The technique was adopted from Hamann et al [26]. Twenty randomly selected microvessels each of 7.5 to 30 μm in diameter of the putamen were measured in each specimen.

### Statistical analysis

Data are presented as mean +/- standard error of mean. Statistical evaluations were performed using t-test.

## Authors' contributions

OU carried out the immunohistochemical experiments, ML performed the statistical analysis and drafted the manuscript. JH participated in the design of the study and collected the brain specimens. AD participated in the study design. GFH supervised the thesis, and participated in its design and coordination.

## Figures and Tables

**Table 1:** Charactistics and cause of death

**Young patients**				**Old non-hypertensive pat.**				**Old hypertensive patients**			
**No**	Age	Cause of death	Sex	**No**	Age	Cause of death	Sex	**No**	Age	Cause of death	Sex
**1**	40	Bronchial carcinoma	f	**12**	68	Lung embolism	f	**20**	81	Gastric hemorrhage	f
**2**	37	Malignant melanoma	f	**13**	67	Lymphoma	F	**21**	70	Pneumonia	f
**3**	27	Lung embolism	f	**14**	82	Mamma-carcinoma	f	**22**	77	Peritonitis	f
**4**	42	Leukemia	f	**15**	71	Lymphoma	F	**23**	74	Resp. insufficiency	m
**5**	34	HIV	f	**16**	77	Liver cirrhosis	f	**24**	72	Bypass surgery	m
**6**	40	Bronchial carcinoma	f	**17**	72	Heart-lung insuff.	m	**25**	74	Hepatorenal syndr.	m
**7**	30	Aplastic anemia	f	**18**	74	Sigmoid-carcinoma	m	**26**	76	Sigmoid-carcinoma	f
**8**	47	Leukemia	m	**19**	74	Stomach-carcinoma	m	**27**	73	Plasmacytoma	f
**9**	44	Aortic aneurysm	m								
**10**	49	Hodgkin' disease	m								
**11**	37	Leukemia	f								
